# Missouri Valley Veterinary Medical Association

**Published:** 1897-01

**Authors:** S. L. Hunter

**Affiliations:** Secretary-Treasurer


### MISSOURI VALLEY VETERINARY MEDICAL ASSOCIATION.

The meeting was convened at Kansas City on Wednesday, October 7, 1896. President Stewart called the meeting to order. Some thirty members were present at the call of the roll. Two new members were admitted to membership.

Owing to the illness of Dr. C. J. Sihler, his paper was not presented.

Dr. R. H. Harrison read a paper entitled "The Human Subject and Animals, their Relationship in Disease." This paper provoked an interesting and instructive discussion.

Dr. L. Enos Day presented a paper for consideration on "Surgical Repair," and Dr. F. W. Hopkins on "Our Profession," both of which were discussed.

President Stewart entertained all present by an interesting account of the Buffalo meeting, reviewing the great work accomplished and its influence for good on future meetings of the Association.

The meeting adjourned after a very profitable session, to meet at the same place on December 9, 1896.

The eleventh regular meeting was held in Kansas City, Mo., Wednesday, December 9, 1896, in Room 23, Masonic building. The meeting was called to order at 7.30 P.M., by President Stewart. The following members responded to roll-call: Drs. Sihler, Barth, Stewart, Harrison, Black, Moore, Kaupp, Day, Hopkins, Bray, and Hunter. Visitors: Drs. Schafter, Allen, Patten, Milnes, and Ovens; and Messrs. Freeman, Cooper, Conrad, Leper, Moore, Pouppirt, Wright, Simpson, and Cowden. The minutes of the last meeting were read and approved.

A communication from Dr. N. S. Mayo was read relating to "Cerebritis in Horses," caused by eating mouldy or wormy corn.

Dr. Schafter was elected to membership.

The committee reported the charges against Dr. J. H. Wattles for unprofessional conduct, being a violation of Article VI., Chapter V., of the Code of Ethics, and, upon motion duly seconded, Dr. Wattles was expelled from the Association.

Excellent papers were read by Drs. C. J. Sihler and Benjamin Kaupp, and were fully and freely discussed.

President Stewart interested the members in the parasite commonly called "Fluke," by a short talk, illustrating the subject by drawings and preserved specimens.

After a most profitable time well spent, the meeting adjourned to meet in Kansas City, Mo., in February, 1897. Essayists for next meeting, Drs. Barth, Schafter, and Harrison.

S. L. HUNTER,
Secretary-Treasurer.

The Ffteenth Annual Meeting of the Michigan State Veterinary Medical Association will be held in Lansing, February 2, 1897. The programme, in addition to Reports of Committees on Intelligence and Education and Diseases, will be added to by a series of operations by various members, among others the "Operation for Roaring," "Spaying a Mare," "Tarsal Tenotomy for Sprain," "Median Neurectomy for Diseases of Fetlock Joint," "Castration of Ridgling Horse."

U. S. V. M. A.—President Osgood has just announced his appointments on the Executive Committee for the ensuing year, as follows: Chairman, W. Horace Hoskins, Leonard Pearson, D. E. Salmon, N. P. Hinkley, L. H. Howard, W. L. Williams, and C. A. Cary.

A movement is on foot to establish a veterinary association in North Carolina. Dr. J. W. Petty, graduate of the National Veterinary College, is actively interested in the movement, and we hope that after its consummation it will be possible to secure legislation for the veterinarians in that State.

Important veterinary meetings—Maine Veterinary Medical Association at Bangor, January, 1897; Chicago Veterinary Society, January 14, 1897; Oneida County Veterinary Medical Society at Rome, February 2, 1897.

